# Destabilization of α-Helical Structure in Solution Improves Bactericidal Activity of Antimicrobial Peptides: Opposite Effects on Bacterial and Viral Targets

**DOI:** 10.1128/AAC.02146-15

**Published:** 2016-03-25

**Authors:** David O. Ulaeto, Christopher J. Morris, Marc A. Fox, Mark Gumbleton, Konrad Beck

**Affiliations:** a CBR Division, Dstl Porton Down, Salisbury, United Kingdom; b School of Pharmacy, University of East Anglia, Norwich, United Kingdom; c College of Biomedical and Life Sciences, School of Pharmacy and Pharmaceutical Sciences, Cardiff University, Cardiff, United Kingdom; d College of Biomedical and Life Sciences, School of Dentistry, Cardiff University, Cardiff, United Kingdom

## Abstract

We have previously examined the mechanism of antimicrobial peptides on the outer membrane of vaccinia virus. We show here that the formulation of peptides LL37 and magainin-2B amide in polysorbate 20 (Tween 20) results in greater reductions in virus titer than formulation without detergent, and the effect is replicated by substitution of polysorbate 20 with high-ionic-strength buffer. In contrast, formulation with polysorbate 20 or high-ionic-strength buffer has the opposite effect on bactericidal activity of both peptides, resulting in lesser reductions in titer for both Gram-positive and Gram-negative bacteria. Circular dichroism spectroscopy shows that the differential action of polysorbate 20 and salt on the virucidal and bactericidal activities correlates with the α-helical content of peptide secondary structure in solution, suggesting that the virucidal and bactericidal activities are mediated through distinct mechanisms. The correlation of a defined structural feature with differential activity against a host-derived viral membrane and the membranes of both Gram-positive and Gram-negative bacteria suggests that the overall helical content in solution under physiological conditions is an important feature for consideration in the design and development of candidate peptide-based antimicrobial compounds.

## INTRODUCTION

Antimicrobial peptides (AMPs) exhibit potent, broad-spectrum bactericidal activity and have been shown to be active against a limited number of viruses (1). They are typically 15 to 40 amino acids in length and variously display the structural characteristics of α-helices, β-sheets, or disordered chains. Some AMPs combine multiple structural elements, and many are stabilized or constrained by intramolecular disulfide bonds or cyclized into circular or loop structures (reviewed in reference 1).

Helical content is an important factor in the microbicidal activity of AMPs, with increased helical content in hydrophobic environments or lipid or detergent micelles seen as a marker for potent microbicidal activity (2–5). For many linear peptides, physiological salt concentrations inhibit microbicidal activity (6, 7). However, α-helical structure of cathelicidin peptides is promoted by elevating the ionic strength of buffers, which correlates with inhibition of antimicrobial activity (8, 9), and there is continuing debate on the mechanistic function of secondary structure for antimicrobial activity. The profound microbicidal activity of AMPs warrants a fuller understanding of their mechanisms of action in order to realize their potential as treatments that avoid resistance mechanisms that apply to conventional antibiotics.

LL37 is a 37-mer α-helical AMP derived posttranslationally from human cathelicidin. It has microbicidal activity against both Gram-positive and Gram-negative bacteria and viruses (10). Studies with multiple primate cathelicidins show that LL37 homologues with lower propensity to adopt α-helical conformation in solution have more potent bactericidal activity, which is less susceptible to inhibition by physiological concentrations of salt (8, 9). LL37 itself will readily adopt α-helical conformation in solution; this is enhanced at physiological salt concentrations, while its bactericidal activity is inhibited.

Salt-dependent conformation change is also a feature of the Xenopus laevis AMP magainin, which adopts a greater degree of α-helicity in solutions containing increased salt, while its bactericidal activity is reduced (3, 11–14). Although magainin has mainly a disordered conformation at physiological salt concentrations, it has been shown to adopt an α-helical conformation in lipid membranes (2, 3, 15). Enhanced α-helical folding in lipid membranes is also observed for LL37 (5), and it is thus possible that whereas adoption of α-helical structure is important for antimicrobial activity of this class of AMP, the point at which the AMP adopts the α-helical structure is equally important, and that preformed helices are less able to disrupt the target membranes.

We have previously shown that LL37 and alanine substituted (S8A, G13A, and G18A) magainin-2 amide (magainin-2B) could inactivate vaccinia virus (VACV) but were unable to inactivate all the virus in a preparation, generally leaving a viable rump of ca. 10% of the starting preparation (16). In this study, we sought to improve the activity of AMPs against VACV by formulation with the nonionic detergent, polysorbate 20 (Tween 20). Experiments were also undertaken with Gram-positive Staphylococcus aureus and Gram-negative Escherichia coli bacteria to determine whether modulation of microbicidal activity applied equally to different types of microbial membranes. We analyzed the effect of the detergent on the secondary structure of LL37 and magainin-2B by circular dichroism (CD) spectroscopy and correlated this with the effect of ionic strength on secondary structure and with the effect of both detergent and ionic strength on activity against S. aureus, E. coli, and VACV.

## MATERIALS AND METHODS

### Reagents.

Unless otherwise stated, all reagents were purchased from Sigma-Aldrich Chemical Company.

### Cell lines and viruses.

Enveloped virions of the IHD-J strain of vaccinia virus were harvested at 18 to 20 h postinfection from the culture supernatant of RK13 rabbit epithelial cells synchronously infected at a multiplicity of infection of ≥10 to provide virions with undamaged envelopes. All cell and virus cultures were maintained in DMEM with 10% fetal bovine serum, 2 mM l-glutamine, and 100 U of penicillin and streptomycin ml^−1^ (tissue culture medium) at 37°C in a 5% CO_2_, humidified atmosphere. Virus was used fresh on the day of preparation and was quantitated by limiting dilution titration on RK13 cells, and the 50% tissue culture infectious dose was calculated by Reed-Muench analysis of virus-positive wells (17, 18). The entire volume of the sample was used in titrations. The requirement for virus to be used as a fresh preparation meant that starting virus titer could not be determined in advance of the experiments and was always determined from control samples as part of the experiment.

### Bacterial strains.

E. coli strain XL1-Blue was obtained from Stratagene, and S. aureus strain ATCC-29213 was obtained from the American Type Culture Collection. For bacterial cultures, a single colony grown on L agar plates (S. aureus) or a loop from a frozen ampoule obtained direct from the supplier (E. coli) was inoculated directly into 5 ml of L broth in a vented T25 tissue culture flask. Two serial dilutions were taken using fresh loops into further T25 flasks containing 5 ml of L broth, and all three flasks were cultured overnight, upright in a shaking incubator at 37°C, after which the most dilute flask with visual turbidity was selected. A sample of the culture was diluted in H_2_O to give an absorbance reading of between 0.01 and 0.02 at 600 nm, and this was used for incubation with AMPs.

After incubation with AMPs for 10 min, bacterial cultures were serially diluted in steps of 1:3, applied in spots to L agar plates (8 spots per dilution, in 10-μl volumes), and cultured overnight at 37°C. After this, each spot was evaluated for bacterial growth, and the data were used to calculate a 50% colony-forming dose by the method of Reed and Muench (17, 18). The entire volume of the sample was used in titrations.

### Peptides.

Peptides were synthesized by Alta Biosciences, Birmingham, United Kingdom. The LL37 sequence used was LLGDFFRKSKEKIGKEFKRIVQRIKDFLRNLVPRTES (19). The magainin-2B sequence used was GIGKFLHAAKKFAKAFVAEIMNS, with the C terminus amidated (11). Unless otherwise stated, peptide stocks were made at 1 mg/ml in phosphate-buffered saline (PBS).

### Peptide treatments.

Peptides were dispensed into tubes at a 10× concentration. Negative controls received an equivalent volume of PBS with no peptide. For treatment in the presence of polysorbate 20 (Tween 20), an appropriate volume of 10× concentration (vol/vol in H_2_O) polysorbate 20 or H_2_O was added to the peptide and control samples and then mixed by vortexing. Finally, the virus or bacteria were added, and the samples were vortexed and incubated at 37°C. Sample volumes were always 50 μl. At the end of the incubation, samples were diluted by the addition of 130 μl of tissue culture medium for virus or 130 μl of L broth for bacteria and then quantitated as described above. In each microbicidal activity experiment, all samples were prepared in triplicate, and all data are presented as means and standard deviations of triplicates.

### CD spectroscopy.

CD spectra were recorded on an Aviv model 215 spectropolarimeter (Aviv Biomedical, Inc., Lakewood, NJ) equipped with a thermostated cell holder. Far-UV spectra (260 to ≤190 nm) were collected in a 0.1-cm quartz cuvette at peptide concentrations of 0.1 to 0.2 mg/ml determined spectroscopically (20). Buffer baselines (with or without polysorbate 20) recorded in the same cell were subtracted, and data were smoothed and normalized to mean residue ellipticities ([ϴ]_MRW_). The instrument was calibrated with camphorsulfonic acid (21). Lyophilized peptides were dissolved in 150 mM NaF and 20 mM KH_2_PO_4_/NaOH (pH 7.4). NaF was used instead of NaCl due to the strong absorbance of chloride ions below λ ≤ 195 nm. Polysorbate 20 in the same buffer was added to peptide samples at least 1 h prior to measurements.

The fraction of α-helical peptide conformation was calculated as described previously (22). Detergent-induced α-helix formation was monitored at 222 nm, and data were fitted assuming a one-site binding mechanism, f^H^ = f_h_^max^ × c/(*K_d_* + *c*), where f^H^ is the fraction of helix, f_h_^max^ is the maximum helix fraction achievable, and *K_d_* is the apparent dissociation constant.

### Statistical analysis.

For statistical analysis, the raw data were transformed by taking the log_10_ of each data-point prior to analysis of variance (ANOVA). The experimental design facilitates the combination of repeat experiments into a single analysis using a two-way ANOVA where one of the parameters is variation between experiments. It is to be expected that in some cases there will be significant variation between repeat experiments because the starting titer of virus and bacterial preparations is not known in advance. However, in all cases where there was a significant variation between experiments, the analysis showed that there was no interaction between the interexperiment variation and the effects, if any, of AMP treatment.

## RESULTS

### Virucidal activity of AMPs is enhanced synergistically by polysorbate 20.

**FIG 1:**
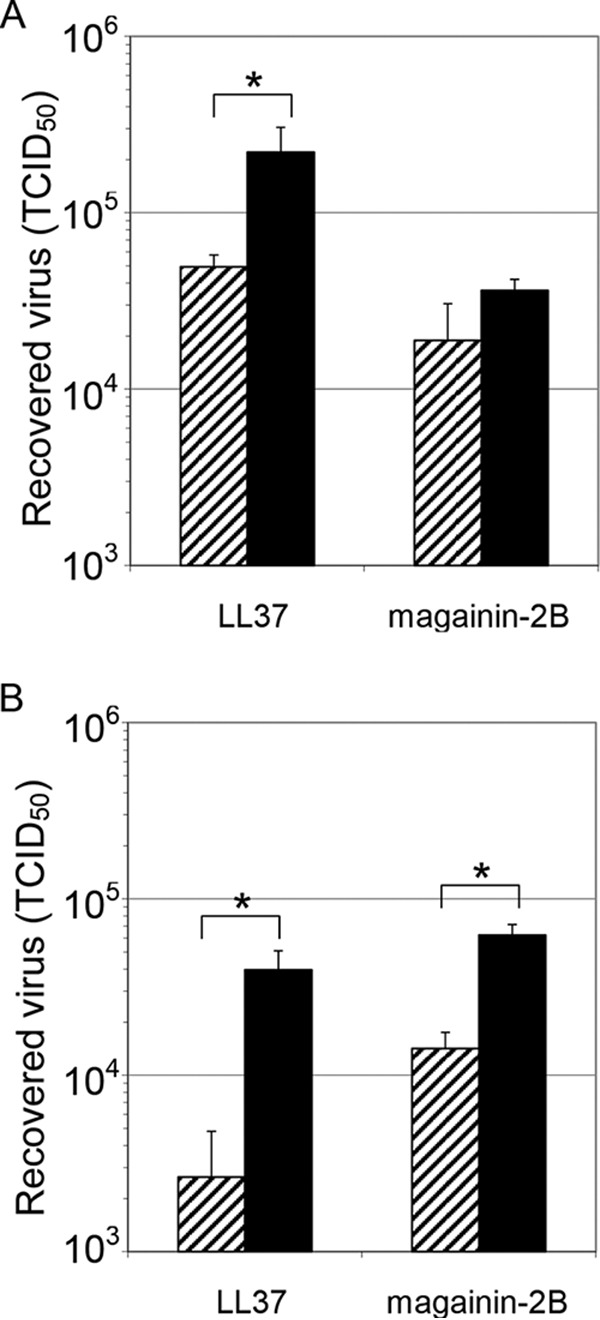
Treatment of VACV preparations with LL37 or magainin-2B. In separate experiments virus preparations in equal volumes were adjusted, to 0 (■) or 100 (▨) μg ml^−1^ LL37 or magainin-2B in the absence (A) or presence (B) of 0.5% (vol/vol) polysorbate 20 for 60 min. Virus was quantitated by Reed-Muench limiting dilution. Data are presented as means and standard deviations of triplicate samples. Each panel is one representative of ≥2 experiments.

We have previously shown that LL37 has greater virucidal activity against VACV than magainin-2B but that both remove the outer membrane of the virus (16). The efficacy of LL37 in inactivating VACV led us to examine methods by which the activity of magainin-2B might be improved. Thus, we took preparations of VACV and treated them with LL37 or magainin-2B in the presence or absence of the nonionic detergent, polysorbate 20, at a concentration above the critical micellar concentration, on the assumption that the detergent would alter the secondary structure of the AMPs. LL37 treatment significantly reduced the quantity of virus recovered (*P* = 0.004) while magainin-2B had a lesser and insignificant effect (*P* = 0.1) in the absence of polysorbate 20 (Fig. 1A). When incubated with virus in the presence of polysorbate 20 for 1 h, magainin-2B acquired significant virucidal activity (<0.001) and a significant enhancement of the activity of LL37 (*P* = 0.004) was also observed (Fig. 1B). Treatment with polysorbate 20 alone for 60 min produced a slight increase in titer, presumably as a result of disaggregation of virus particles (data not shown).

**FIG 2:**
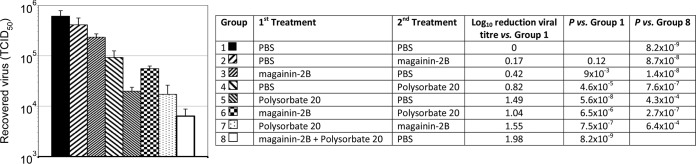
Treatment of VACV preparations with magainin-2B in the presence and absence of polysorbate 20. Virus preparations were divided into 24 equal aliquots and treated sequentially with PBS, followed by PBS; PBS, followed by magainin-2B; magainin-2B, followed by PBS; PBS, followed by polysorbate 20; polysorbate 20, followed by PBS; magainin-2B, followed by polysorbate 20; polysorbate 20, followed by magainin-2B; or magainin-2B and polysorbate 20 together, followed by PBS (groups 1 to 8, respectively, in the figure). Magainin-2B was used at 100 μg ml^−1^, and polysorbate 20 was used at 0.5% (vol/vol). Sequential incubations were for 90 min each, with the virus being washed twice in between the two treatments. After the second wash, the virus was resuspended directly in the second treatment in 50-μl volumes. After the second treatment, the samples were adjusted to 200 μl with tissue culture medium and subjected to manual Potter-Elvejhem homogenization and titration without additional washes. Data are presented as means ± the standard deviations of triplicate samples. The results of one representative experiment of two experiments are shown.

The improved activity of both LL37 and magainin-2B against VACV in the presence of polysorbate 20 led us to determine whether the AMP and the detergent were acting independently or cooperatively. Virus was treated with magainin-2B and subsequently with polysorbate 20 after first washing away the AMP or vice versa. To control for the necessity of incubating treated controls for a further 90 min after the first treatment, replicate samples were treated with polysorbate 20 for 90 min, followed by PBS for 90 min, with a parallel group treated with PBS for 90 min, followed by polysorbate 20 for 90 min. A similar approach was taken with the magainin-2B controls. As expected, both treatment with PBS followed by magainin-2B (*P* = 0.123) and magainin-2B followed by PBS (*P* = 0.009) produced a slight effect, although only in the latter case was this significant. Polysorbate 20 alone produced an unexpected significant reduction in virus titer when the first incubation was in PBS (*P* = 4.61 × 10^−5^), possibly as a result of increasing the incubation time to 90 min and a nonspecific detergent-like effect on the viral membrane. This was even more apparent when the polysorbate 20 treatment preceded the PBS treatment (*P* = 5.64 × 10^−8^), and the difference between polysorbate 20 as the first treatment and polysorbate 20 as the second treatment in these control samples was significant (*P* = 2.17 × 10^−5^) (Fig. 2). This is likely to reflect a time-dependent degradation of virus membranes by the detergent and fits with previous observations demonstrating the time-dependent effect of polysorbate 20 on the density of VACV virions (16).

Where samples were sequentially treated with magainin-2B and polysorbate 20, or vice versa, treatment with detergent first gave a level of virus inactivation that was similar to treatment with detergent first and then subsequently with PBS. Treatment with magainin-2B first yielded a level of inactivation that was similar to that seen when treating with PBS first and then subsequently with detergent. This indicates that treatment with AMP and detergent sequentially is equivalent to omitting the AMP. Importantly, treatment with magainin-2B and polysorbate 20 in combination, followed by a subsequent treatment with PBS, yielded a level of inactivation that was significantly greater than all other combinations (*P* values ranged from 4.32 × 10^−4^ to 8.67 × 10^−8^) (Fig. 2). This suggests that magainin-2B and polysorbate 20 may act synergistically to inactivate VACV and that the effect on VACV of either in isolation does not render the virus particles more susceptible to the other in isolation.

**FIG 3:**
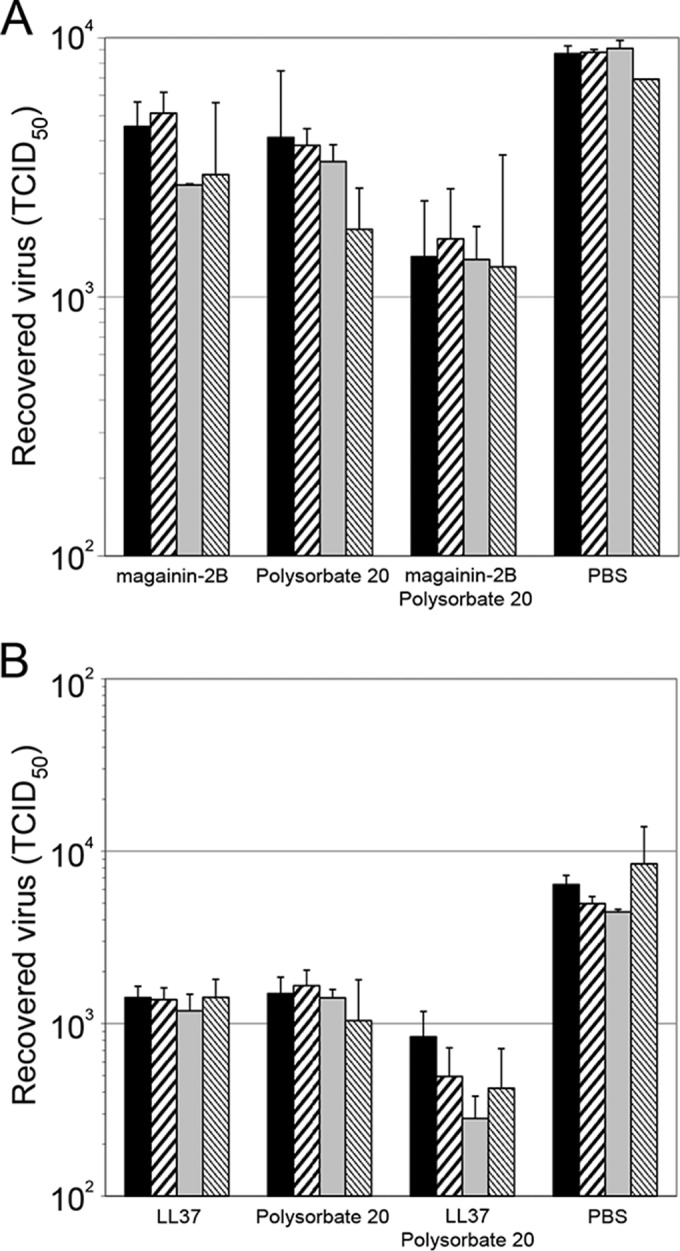
Time course of AMP and polysorbate 20 activity against VACV. Virus preparations were divided into equal aliquots and adjusted to 0 or 100 μg ml^−1^ magainin-2B (A) or LL37 (B) and/or 0 or 0.5% (vol/vol) polysorbate 20 and incubated at 37°C for 30 (■), 60 (▨), 90 (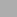), or 120 (▧) min. Virus was quantitated by Reed-Muench limiting dilution. Data are presented as means ± the standard deviations of triplicate samples. The results of one representative experiment of two experiments are shown.

The observation of virucidal activity for polysorbate 20 after a 90-min incubation, while a 60-min incubation did not reduce virus titers in previous experiments (data not shown), suggested that the effects of both detergent and AMP might be time dependent. Accordingly, we examined the effects of polysorbate 20 and magainin-2B or LL37 separately and in combination over a range of incubation times. Magainin-2B was time dependent in its effect, taking more than 60 min to inactivate a significant proportion of the virus in the sample relative to the PBS controls (*P* = 0.003) (Fig. 3A). The effect on LL37 was not time dependent in these experiments, which may reflect the greater virucidal efficacy of this AMP for VACV (Fig. 3B). The time dependency of polysorbate 20 virucidal activity varied considerably between experiments. However, in all experiments when detergent and either AMP were combined, an enhanced effect was seen after a 30- or 60-min incubation, relative to PBS, polysorbate 20 alone, or AMP alone (*P* values ranged from 1.1 × 10^−5^ to 2.4 × 10^−12^), providing further evidence of a synergistic interaction between the two.

Synergism between AMP and polysorbate 20 suggests that the effect is mediated by alteration of the AMP secondary structure in solution, prior to AMP interaction with the target membrane. This has implications for the future development of AMPs as potential anti-infective agents, since it demonstrates that formulation in aqueous solution can significantly affect their activity.

### Polysorbate 20 increases the α-helical content of AMPs in aqueous solution.

**FIG 4:**
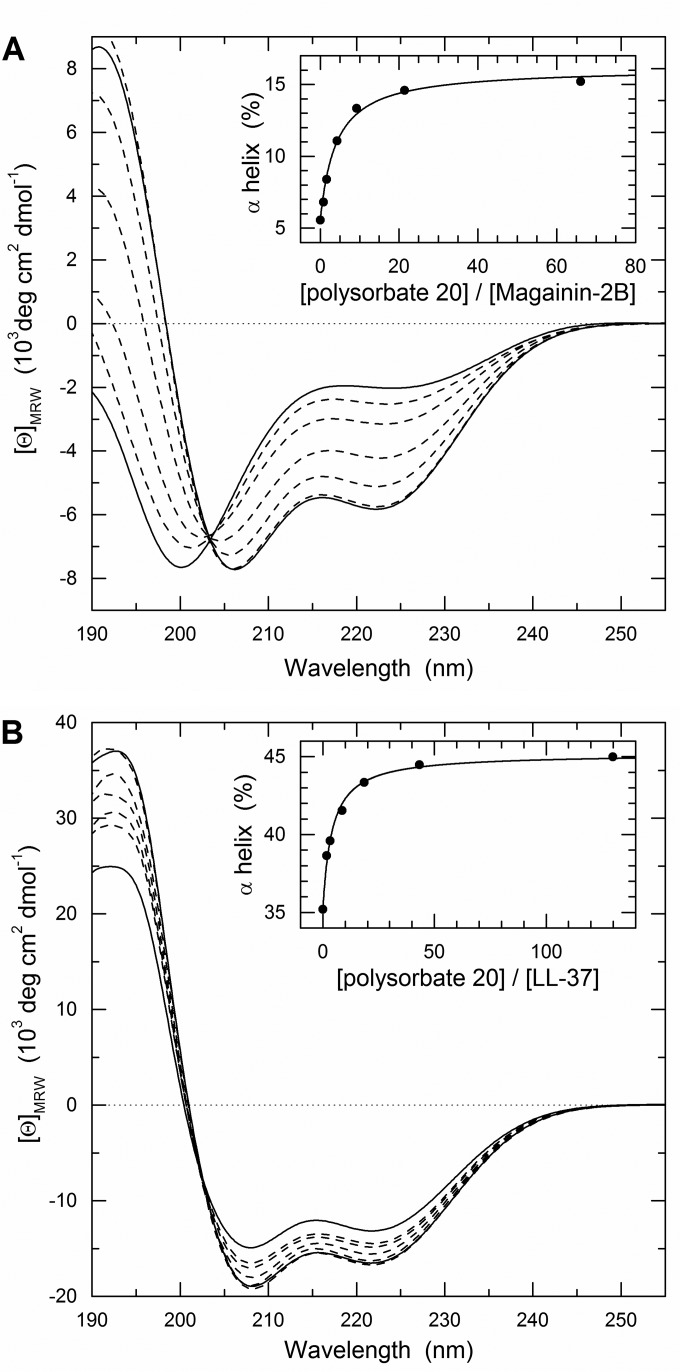
CD spectroscopy. Far-UV CD spectra were recorded for magainin-2B (A) and LL37 (B) in the absence or presence of 0.40% polysorbate 20 (solid lines) at 37°C. Dashed lines represent spectra recorded at detergent concentrations (from the top to the bottom at 220 nm) of 0.01, 0.02, 0.05, 0.10, and 0.20% (vol/vol). Insets show the degree of α-helicity derived from the [Θ]_222_ plotted as a function of the molar detergent/peptide ratios.

The apparent synergy between AMPs and polysorbate 20 for inactivation of VACV led us to examine the secondary structure of the AMPs in the presence or absence of polysorbate 20 by CD spectroscopy. In salt-containing buffer, the magainin-2B CD spectrum shows the characteristics of an unordered structure with a pronounced minimum at ∼200 nm and a shoulder of negative ellipticity at ∼227 nm (Fig. 4A). These characteristics are also observed at 2°C, at which the magnitude of the signal is about 1.5 times greater (data not shown). In the presence of 0.4% (vol/vol) polysorbate 20, the spectrum shows minima at 208 and 222 nm and a positive maximum at 192 nm, which are the characteristic CD bands for an α-helical conformation. Spectra recorded upon titration of polysorbate 20 (from 0.01 to 0.40%) exhibit a single isodichroic point at 203 nm, which is compatible with the assumption that the coil-to-helix conversion occurs as a two-state process. Data fitting results in an apparent *K_d_* = 0.48 ± 0.09 mM with a maximum α-helix content of 16.9 ± 0.7%. Half-maximal change in helicity is found at a 4.1:1 detergent/peptide ratio corresponding to ∼0.05% polysorbate 20 (Fig. 4A, inset).

The CD spectrum of LL-37 in salt-containing buffer is indicative for a pronounced α-helical conformation (Fig. 4B), whereas in the absence of salt it shows the characteristics of an unordered structure (23). The calculated α-helix content of 35% at 37°C is only slightly lower than the 38% observed at 2°C. The spectra are in quantitative agreement with those reported earlier (23). Upon addition of polysorbate 20, the CD intensities increase, reaching half-saturation at a detergent/LL37 ratio of ∼4.1 ± 0.5 corresponding to a *K_d_* = 0.23 ± 0.03 mM and a maximal α-helix content of 45.5% ± 0.3% (Fig. 4B, inset). Also in this case, spectra recorded at increasing polysorbate 20 concentrations show a single isodichroic point at 203 nm.

It is already known that AMPs adopt α-helical conformation in target membranes. Our findings suggest that, at least for VACV, it is important that α-helical conformation is achieved before the AMP comes into contact with the membrane. It is interesting that the effect of polysorbate 20 on α-helical content of the AMP parallels that of elevated ionic strength buffer, which is known to inhibit bactericidal activity of AMPs. This suggests that the enhanced activity observed against VACV might not apply to bacterial targets.

### The bactericidal activity of AMPs is attenuated by polysorbate 20 or elevated ionic strength.

**FIG 5:**
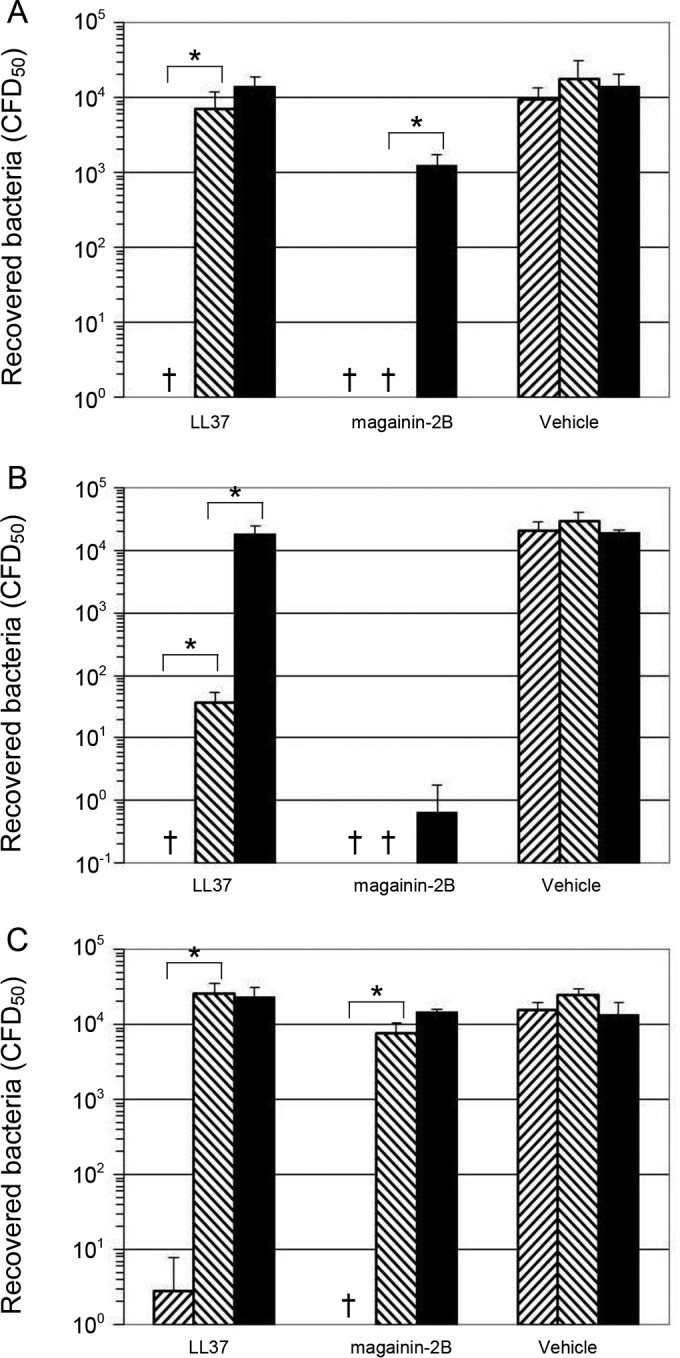
Attenuation of AMP antibacterial activity by NaF and NaCl. E. coli (A and B) or S. aureus (C) bacteria were incubated with either LL37 or magainin-2B amide at a concentration of 100 μg ml^−1^ for 10 min in H_2_O. Peptides and vehicle (1/10 dilution of PBS in H_2_O) controls were adjusted to either 500 (■), 250 (▧), or 0 (▨) mM NaF (B and C) or 500 (■), 250 (▧), or 11 (▨) mM NaCl (A) before the addition of bacteria. After incubation with AMP for 10 min at room temperature, samples were serially diluted in L broth and plated on L agar plates, followed by incubation overnight at 37°C. After this, the colonies were enumerated for 50% CFU using the Reed-Muench method. †, No colonies were recovered (100% kill).

**FIG 6:**
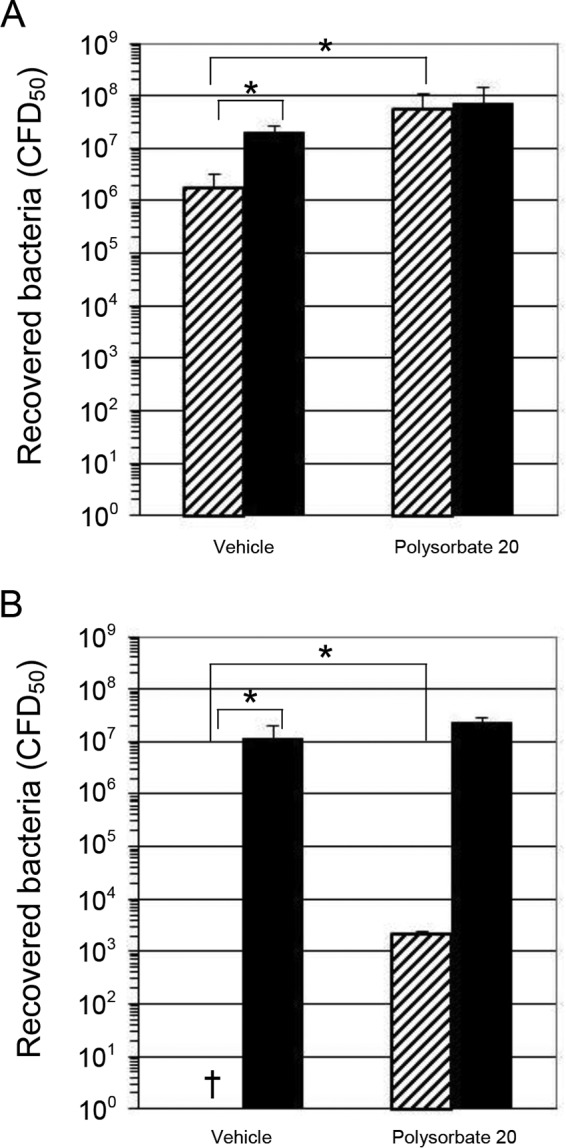
Attenuation of AMP antibacterial activity by polysorbate 20. E. coli bacteria were incubated with either LL37 (A) or magainin-2B amide (B) at a concentration of 100 μg ml^−1^ for 10 min in L broth. Peptides (▨) and vehicle (1/10 dilution of PBS in H_2_O) controls (■) were adjusted to either 0.5% polysorbate 20 or an equal amount of diluent (H_2_O) before the addition of bacteria. After incubation with antimicrobial peptide for 10 min at room temperature, the samples were serially diluted in L broth and plated on L agar plates, followed by incubation overnight at 37°C. After this, the colonies were enumerated for 50% CFU using the Reed-Muench method. †, No colonies were recovered (100% kill). The vehicle controls were PBS, added to a final dilution of 1:10 (vol/vol). The results of one representative experiment of two experiments are shown.

The parallel effects of increasing ionic strength and increasing detergent concentration on AMP α-helix content led us to examine the effect of polysorbate 20 on bactericidal activity of the AMPs. The salt-dependent inhibition of AMP bactericidal activity shown by others was confirmed with E. coli bacteria treated in the presence of increasing concentrations of NaF (to mirror conditions used in CD spectroscopy) and NaCl, with a marked inhibition of bactericidal activity at high ionic strength (*P* values ranged from 5.7 × 10^−9^ to 3.7 × 10^−11^, except for magainin-2B in the presence of NaCl, where salt concentration did not affect AMP activity, and *P* = 0.56) (Fig. 5A and B). The effect of NaF was also examined for S. aureus, with a similar result (*P* values of 7.6 × 10^−9^ and 2.1 × 10^−12^) (Fig. 5C). When E. coli experiments were repeated with polysorbate 20 instead of salt, an effect opposite that seen with VACV and similar to that seen against bacteria in the presence of salt was observed (Fig. 6). The bactericidal activity of the AMPs was reduced by formulation with polysorbate 20 (*P* = 0.03 for LL37 and *P* = 9.8 × 10^−9^ for magainin-2B).

These data show correlation of the effects of polysorbate 20 and salt on the bactericidal activity of AMPs just as the effects of polysorbate 20 and salt on AMP secondary structure in aqueous solution are correlated. The correlations suggest a mechanistic relationship between the observed secondary structure and the levels of bactericidal activity.

### The virucidal activity of AMPs is enhanced by elevated ionic strength.

**FIG 7:**
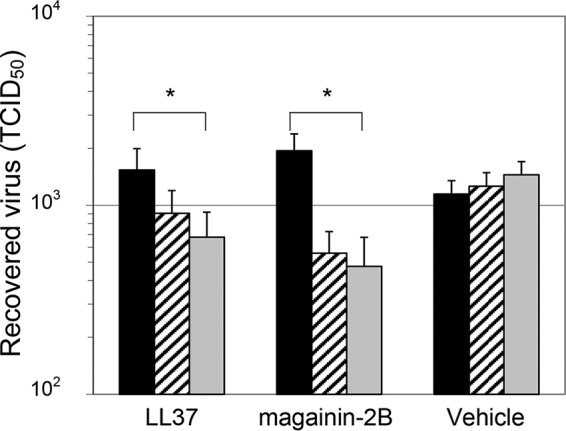
Enhancement of AMP virucidal activity by NaF. VACV preparations were divided into equal aliquots and adjusted 0 or 100 μg ml^−1^ LL37 or magainin-2B, and 0 mM/(■), 250 mM (▨), or 500 mM (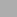) NaF, followed by incubation at 37°C for 60 min. Vehicle controls were a 1/10 dilution of PBS in H_2_O. Virus was quantitated by Reed-Muench limiting dilution. Data are presented as means and standard deviations of triplicate samples. The results of one representative experiment of two experiments are shown.

When virucidal activity of the AMPs was assessed in the presence of NaF, an enhancement of activity was observed analogous to the situation with virucidal activity in the presence of polysorbate 20 and opposite the effect observed in the treatment of bacteria (*P* = 0.03 for LL37 and *P* = 0.003 for magainin-2B) (Fig. 7). This further correlates the effects of polysorbate 20 and salt on virucidal activity; the differential direction of potentiation against bacteria and VACV is also correlated, strengthening the interpretation that the AMP secondary structure in aqueous solution is important for membrane attack.

Potentiation of virucidal activity by elevated ionic strength was also examined for a second virus, Semliki Forest virus (SFV), of the alphavirus genus of the family Togaviridae. However, neither LL37 nor magainin-2B had any activity against SFV either in high- or low-ionic-strength buffer (see Fig. S1 in the supplemental material). Polysorbate 20 was not included in the SFV experiments since neither AMP had baseline activity against the virus and polysorbate 20 inactivates the related Venezuelan equine encephalitis virus by itself (D. Ulaeto, unpublished data). There is much less data in the literature on the effect of AMPs on viruses, suggesting a general lower susceptibility of enveloped viruses to inactivation by AMPs. Interestingly, unlike SFV, which obtains its envelope from the plasma membrane, VACV obtains its outer envelope from the Golgi apparatus. The failure of the AMPs to degrade SFV does not alter our interpretation of the data with bacteria and VACV, demonstrating opposite effects of altered secondary structure on microbicidal activity of the AMPs on the two types of membrane. This is highly suggestive of multiple, distinct modes of attack residing in the same peptide.

## DISCUSSION

Elevated-ionic-strength buffer is known to attenuate the bactericidal activity of AMPs (6, 7). We have confirmed this and demonstrated in the same system that polysorbate 20 has the same effect of attenuating activity. Our interpretation is that the modulation mediated by polysorbate 20 and salt is likely to be due to their stabilization of α-helical content in solution.

The VACV envelope is derived from the host *trans*-Golgi network and is thus zwitterionic, unlike the bacterial membranes of E. coli and S. aureus, which are anionic. This would be expected to influence the interaction with AMPs, which generally carry a net positive charge (LL37, +6; magainin-2B, +4) (4). Small linear AMPs are often largely unstructured in water, although many will adopt some α-helical structure in ionic buffer solutions and, in some cases, those with a greater propensity to form an α-helix in solution are able to more aggressively attack eukaryotic membranes (8, 9). To bind and insert into microbial membranes, unfolded AMPs like the magainins are thought to interact mainly via electrostatic and/or hydrophobic interactions with the lipid bilayer. Insertion of the nonpolar side chains into the hydrophobic core of the membrane is accompanied by a structural conversion to a helical conformation and depolarization of the membrane through the formation of pores (24, 25). In contrast to magainin-2B, LL-37 has a significant degree of α-helix in solutions that mimic physiological salt conditions (23), probably due to the amphipathic character of residues 11 to 32. This region contains three heptad repeats of the form (*abcdefg*)_n_ with hydrophobic residues occupying positions *a* and *d*; thus, this region could serve as an assembly site for forming an α-helical coiled coil (26). It is interesting to speculate that the different degrees of α-helix of LL-37 and magainin-2B in physiological buffers may be responsible for the differential activity of the AMPs in physiological buffer in the absence of polysorbate 20, whereby LL37 in physiological buffer has greater activity against VACV than magainin-2B, whereas the reverse is true for activity against E. coli and S. aureus.

**FIG 8:**
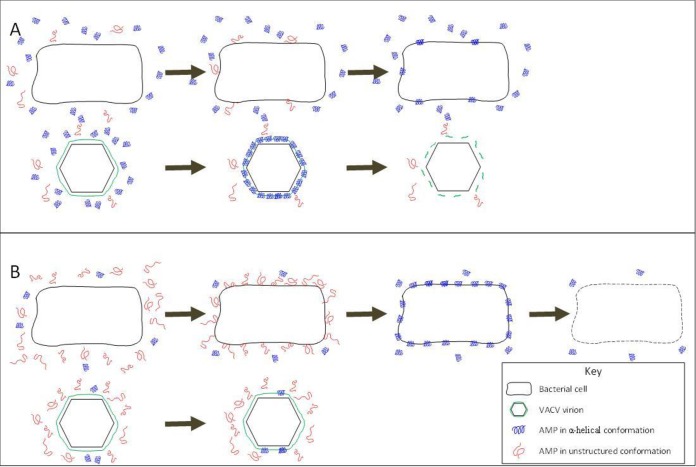
Schematic representation of the influence of secondary structure of a linear AMP on bacterial and viral membrane interactions. (A) In a helix-promoting formulation such as polysorbate 20 or elevated ionic strength, the AMP secondary structure is biased toward adopting an α-helix. The helical AMP readily associates with the virus membrane, causing it to fragment. The helical AMP does not readily interact with the bacterial membrane, leaving the bacterium still viable. (B) In a helix-resisting formulation, such as low-ionic-strength buffer, the AMP secondary structure is biased toward unstructured conformation. The unstructured AMP readily associates with the bacterial membrane, converting to a helical conformation in the membrane and forming depolarizing pores. The unstructured AMP does not readily interact with the virus membrane leaving the virus still viable.

Our results showing that polysorbate 20 modulates AMP activity against bacteria and VACV in opposite directions are a strong indication that AMPs act on the different targets by different mechanisms. The correlation of the effects of polysorbate 20 and ionic strength on (i) AMP secondary structure, (ii) bactericidal activity, and (iii) virucidal activity suggest that the relationship between secondary structure in aqueous solution and microbicidal activity is causal. The data further suggest that for optimal bactericidal activity the AMP should adopt unstructured conformation prior to interaction with the target membrane, and that for optimal virucidal activity it should adopt α-helical conformation prior to interaction with the target membrane (Fig. 8). Although high α-helical content is known to be an important feature of bactericidal AMPs (8, 9, 27, 28), prefolding into an α-helix in solution may attenuate the ability to insert into both Gram-positive and Gram-negative bacterial membranes, but enhance the ability to insert into some eukaryotic membranes. In essence, the secondary structure of the AMP in solution can be seen as increasing or decreasing the effective concentration of AMP for a given target membrane without altering the absolute concentration in the preparation. Previous work has shown that linear AMPs degrade VACV membranes by a carpet-based mechanism (16), whereas bactericidal activity is more commonly ascribed to pore formation (24, 25). Our data indicate that a single peptide may act by more than one mode of membrane attack. It seems possible that a carpet mechanism and pore formation are differentially favored under conditions such as those we have used and, conceivably, are differentially effective against the different types of target membrane.

This study suggests that formulation as an aqueous solution will present difficulties for the clinical development of AMPs for treatment of systemic infections. However, we have described a structural requirement for AMP activity prior to association with a target membrane and how preformed secondary structure can affect selectivity for target membranes. This will facilitate wider efforts to develop complex formulations to maintain desirable secondary structure after systemic administration.

## Supplementary Material

Supplemental material
