# Supplementary material for: Destabilization of α-Helical Structure in Solution Improves Bactericidal Activity of Antimicrobial Peptides: Opposite Effects on Bacterial and Viral Targets
Source: Antimicrob Agents Chemother. 2016 Mar 25;60(4):1984–91. doi: 10.1128/AAC.02146-15 (PMC4808201; doi:10.1128/AAC.02146-15)
Supplement: Supplemental material [file supp_60_4_1984__index.html]

Destabilization of α-Helical Structure in Solution Improves Bactericidal Activity of Antimicrobial Peptides: Opposite Effects on Bacterial and Viral Targets — Supplemental material 

# Destabilization of α-Helical Structure in Solution Improves Bactericidal Activity of Antimicrobial Peptides: Opposite Effects on Bacterial and Viral Targets

## Supplemental material

- Supplemental file 1 -

  Supplemental Figure S1: effect on AMP virucidal activity of NaF against Semliki Forest virus.

  PDF, 228K
