# Supplementary material for: Destabilization of α-Helical Structure in Solution Improves Bactericidal Activity of Antimicrobial Peptides: Opposite Effects on Bacterial and Viral Targets
Source: Antimicrob Agents Chemother. 2016 Mar 25;60(4):1984–91. doi: 10.1128/AAC.02146-15 (PMC4808201; doi:10.1128/AAC.02146-15)
Supplement: Supplemental material [file AAC.02146-15_zac003164974so1.pdf]

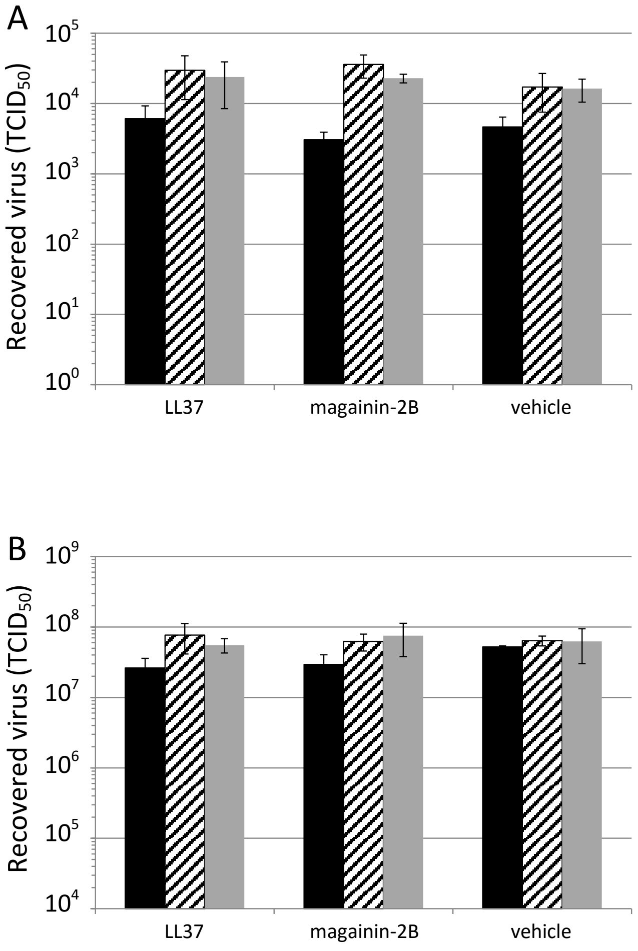

SUPPLEMENTARY FIGURE 1. Effect on AMP virucidal activity of NaF against Semliki firest virus (SFV). SFV preparations were divided into equal aliquots and adjusted to 0 or 100 μg ml^{-1} LL37 or magainin-2B, and 0 mM (■), 250 mM (▨) or 500 mM (□) NaF, and incubated at 37˚C for 60 minutes. Vehicle controls were 1/10 dilution of PBS in H_2O. Virus was quantitated by Reed-Muench limiting dilution. Data is presented as means and standard deviations of triplicate samples. Two independent experiments using different starting concentrations of virus are shown in panels A and B. Statistical analysis was by two-way ANOVA of log_{10} transformed data from the combined experiments. No significant difference was observed between AMP treated and untreated samples (LL37, P = 0.76; Magainin 2B, P =0.09).
